# Whole genome sequencing reveals high differentiation, low levels of genetic diversity and short runs of homozygosity among Swedish wels catfish

**DOI:** 10.1038/s41437-021-00438-5

**Published:** 2021-05-07

**Authors:** Axel Jensen, Mette Lillie, Kristofer Bergström, Per Larsson, Jacob Höglund

**Affiliations:** 1 Department of Ecology and Genetics, Animal Ecology, Uppsala University, Uppsala, Sweden; 2 Department of Biological and Environmental Sciences, University of Gothenburg, Gothenburg, Sweden; 3 Department of Biology and Environmental Science, Faculty of Health and Life Sciences, Linnaeus University, Kalmar, Sweden

**Keywords:** Ecology, Genetics

## Abstract

The use of genetic markers in the context of conservation is largely being outcompeted by whole-genome data. Comparative studies between the two are sparse, and the knowledge about potential effects of this methodology shift is limited. Here, we used whole-genome sequencing data to assess the genetic status of peripheral populations of the wels catfish (*Silurus glanis*), and discuss the results in light of a recent microsatellite study of the same populations. The Swedish populations of the wels catfish have suffered from severe declines during the last centuries and persists in only a few isolated water systems. Fragmented populations generally are at greater risk of extinction, for example due to loss of genetic diversity, and may thus require conservation actions. We sequenced individuals from the three remaining native populations (Båven, Emån, and Möckeln) and one reintroduced population of admixed origin (Helge å), and found that genetic diversity was highest in Emån but low overall, with strong differentiation among the populations. No signature of recent inbreeding was found, but a considerable number of short runs of homozygosity were present in all populations, likely linked to historically small population sizes and bottleneck events. Genetic substructure within any of the native populations was at best weak. Individuals from the admixed population Helge å shared most genetic ancestry with the Båven population (72%). Our results are largely in agreement with the microsatellite study, and stresses the need to protect these isolated populations at the northern edge of the distribution of the species.

## Introduction

Since genomic techniques have become more affordable and available, the field of conservation genetics has moved to conservation genomics (Allendorf 2017). It is now feasible to collect polymorphism data for the whole genome at moderate cost and effort (Hendricks et al. 2018). Already there has been debate how the new methods, techniques, and results may be implemented in practical conservation (Allendorf et al. 2010; Garner et al. 2016; McMahon et al. 2014; Shafer et al. 2015; Shafer et al. 2016). Conservation genetic studies typically allow inference of genetic diversity within populations and population structure and differentiation among populations within species (Allendorf and Luikart 2007; Höglund 2009). Genetic markers have also been used to study the extent of inbreeding (Frankham et al. 2010). With genomic data, such estimates are readily available and become arguably more accurate as larger portions of the genome are covered. Whether genomic-based estimates alter interpretations and downstream recommendations is still not well-studied, but is certainly a pertinent question to conservation biology.

Following the revolution in high throughput sequencing techniques, new possibilities for empirically studying populations and conservation genetics with much higher resolution has opened up (Shendure and Ji 2008). The possibility to sequence a complete genome at a reasonable cost has made studies based on whole-genome sequencing data increasingly common (e.g., Begun et al. 2007; Lamichhaney et al. 2017), typically with a focus on single nucleotide polymorphism (SNP) datasets, to assess genetic variation and differentiation. The fact that hundreds of thousands, or even millions of SNPs can be analyzed within a single individual makes them excellent as markers of population structure and gene flow, providing diverse opportunities for genetic analyses (Helyar et al. 2011).

Populations at the periphery of a distribution constitute interesting case studies for conservation (e.g., Bylak and Kukuła 2018, 2020). Compared to populations at the core of the distribution range, peripheral populations may be adapted to different selective pressures and, thus, hold important evolutionary potential in a rapidly changing environment (Channell and Lomolino 2000; Channell 2004). Such populations are also typically small, fragmented, may have suffered loss of genetic variation, and are consequently often threatened by local extinction (reviewed by e.g., Höglund 2009; Allendorf et al. 2013). Being small and fragmented (Channell 2004), allele frequency changes in such populations are predicted to be primarily determined by genetic drift, while the force of selection is of minor importance (Kimura 1983). Data from many loci distributed over the entire genome of a study organism can provide a robust framework for determining the relative importance of drift versus selection.

**Fig. 1:**
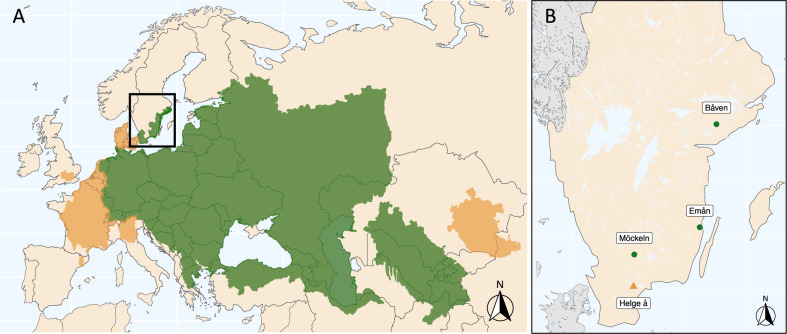
Distribution of the wels catfish, *Silurus glanis*. **A** Native (green) and introduced (orange) distribution range of wels catfish globally, adapted from IUCN (Freyhof 2010). **B** The locations of the only three remaining native populations of wels catfish in Sweden (green circles), as well as of the sampled reintroduced population (orange triangle). In addition, one introduced population, not marked on the map, is present just north of the native population of Emån (not sampled in this study).

The wels catfish *Silurus glanis* is a teleost fish in the order Siluriformes that inhabits freshwater lakes and rivers. Its native range spans from central Europe to western Russia and from southern Scandinavia to northern Iran (Copp et al. 2009) (Fig. 1). Globally, it is considered as of least concern by the IUCN (Freyhof 2010) and is even an invasive species in some western European countries where it has been introduced for angling purposes (Cucherousset et al. 2018; Guillerault et al. 2015). The species natural distribution resulted from an expansion after the last glacial period from a single glacial refugium, located in the Ponto-Caspian region in eastern Europe (Triantafyllidis et al. 2002). Sweden was likely colonized ~8000–9500 years ago, when the Baltic sea basin was a large freshwater lake, disconnected from the oceans (Nathanson 1987).

The wels catfish was historically found across southeastern Sweden, but since the 1800s a dramatic population decline has occurred (Nathanson 1987). In the late 1900s, the species was considered on the verge of extinction and was categorized as *critically endangered* on the Swedish red list (ArtDatabanken 2020). Since then, indications of population increase (Borger and Kjellberg 2006; Havs-och-vattenmyndighetens 2017) have led to the present red list category of *near threatened* (ArtDatabanken 2020). Lake Båven, Lake Möckeln, and the Em River (referred to hereafter as Emån), along with their connected water systems, are the only localities with extant native populations (Fig. 1). In addition, re-introductions have established two currently self-sustaining populations, including one in the lower parts of the Helge å River, which is included in our study. Individuals deriving from all three native populations have been used as stock during re-introductions to the Helge å River (Svensson et al. 2013).

Located at the periphery of the species’ northern distribution range, the Swedish populations may be particularly vulnerable to the effects of genetic drift and bottlenecks (Eckert et al. 2008). Populations at range limits may also hold unique evolutionary potential due to their divergence from central populations (Lesica and Allendorf 1995). High levels of genetic differentiation have previously been reported across catfish populations in Europe using a variety of genetic markers, including allozyme markers (Triantafyllidis et al. 1999a), mitochondrial markers (Triantafyllidis et al. 1999b), mitochondrial sequence haplotypes (Krieg et al. 2000), and nuclear microsatellite markers (Triantafyllidis et al. 2002). Studies demonstrated a general pattern of higher genetic diversity in the eastern part of the range (close to the putative glacial refuge) (Krieg et al. 2000; Triantafyllidis et al. 2002) compared to populations from the southern range limit in Greece which displayed lower diversity as well as a population from the western range limit, Lake Morat in Switzerland, where the lowest genetic diversity of all studied populations was found (Triantafyllidis et al. 2002).

Microsatellite markers applied in Triantafyllidis et al. (2002) have recently been used to infer population substructure and diversity in Sweden (Palm et al. 2019). Genetic diversity was among the lowest reported in Europe, and high levels of genetic differentiation were observed between the Swedish populations (Palm et al. 2019). Among the three native populations, no substantial differences in genetic diversity were apparent. Båven had slightly higher expected heterozygosity (*H*_E_) than Emån and Möckeln, while the number of alleles per locus was lowest in Båven and highest in Emån. Population differentiation (*F*_ST_) estimates were high in all comparisons (0.34–0.57).

Here, we sequence and present a de-novo assembly of the wels catfish genome. In addition, we use whole-genome resequencing of individuals from four Swedish populations (three native and one reintroduced) to make inferences on genetic diversity, differentiation, and estimates of inbreeding in Swedish catfish. We use our data to compare with similar estimates obtained in a previously published genetic study using microsatellites (Palm et al. 2019) on the same populations, to determine whether our whole-genome approach yields additional population genetic inferences and conservation genetic recommendations.

## Materials and methods

### Assembly of de novo genome

A blood sample was collected from a wels catfish from the Emån population (GPS 57.142183, 16.455940) (Table S1) into an EDTA blood collection tube and stored on ice for ~4 h during transport. High molecular weight DNA was then immediately purified using the Qiagen MagAttract HMW DNA kit (Qiagen, Hilden, Germany), following the manufacturer’s standard protocol. The SNP&SEQ Technology Platform in Uppsala carried out DNA quantity and quality assessment, and library preparation for 10X Chromium linked-read sequencing (10xGenomics 2020). The library was sequenced on a NovaSeq SP (2 × 150 bp) by the SNP&SEQ Technology Platform in Uppsala. The resulting linked-reads were assembled using Supernova v. 2.1.1 “run” (Weisenfeld et al.^ 2017^) with a maximum of 520 million reads (--maxreads 520), equivalent to 78 X coverage. Fasta format of the assembly was generated by supernova “mkoutput” for pseudohap2, which generates two-phased haplotypes; the first of which was used as reference genome in subsequent analyses.

### Whole-genome resequencing

A total of 66 samples were collected for whole-genome sequencing, including 20 from each native population (Båven: GPS 59.014207, 16.932581; Emån: GPS 57.142183, 16.455940; and Möckeln: GPS 56.673406, 14.190471) and six from the reintroduced, admixed population of Helge å (GPS 56.034165, 14.135506) (Sampling details listed in Table S1; Finer scale distribution maps, highlighting waterways, in Fig. S1). The samples consisted of fin tissues from unique individuals that were collected in 2018 and stored in ethanol. DNA was extracted from these tissues using the Qiagen DNeasy Blood & Tissue kit (Qiagen, Hilden, Germany) following the manufacturer’s standard protocol.

Paired-end sequencing libraries were constructed using the Nextera DNA Flex Library Prep Kit (Illumina, San Diego, CA, USA), following the provided standard protocol, with Nextera DNA CD Indexes to uniquely index each sample prior to pooling. Library quality was assessed using the High Sensitivity DNA Kit for the Agilent 2100 Bioanalyzer electrophoresis system (Agilent Technologies, Santa Clara, CA, USA). In the pooling step, an equal volume from each native sample was pooled (theoretical sequencing coverage approximated to ~ 12 ×). Pool quality and concentration were assessed using both a Bioanalyzer High Sensitivity DNA Kit and a Qubit 2.0 fluorometer (Invitrogen, Carlsbad, CA, USA) dsDNA HS (High Sensitivity) Assay Kit. Library quality of the Helge å samples was lower than that observed for native samples. Therefore, Helge å samples were added to the sequencing pool to give an approximate theoretical 5 × coverage. This final pool, containing 66 individually indexed samples, was sequenced on one S4 lane (150 bp paired-end reads) of an Illumina Novaseq 6000 by the SNP&SEQ Technology Platform in Uppsala.

### Variant calling

After quality control in FastQC (Andrews 2010), the sequenced reads were converted from fastq format to sam format and Illumina adapters were marked using Picard v.2.20.4 (http://broadinstitute.github.io/picard), resulting in an unmapped BAM file. Reads were mapped to our de novo genome assembly using bwa mem v.0.7.17 (Li and Durbin 2009). Mapped bams were sorted and duplicates marked by Picard, then they were subsequently indexed by SAMtools v.1.10 (Li et al. 2009), ready for variant calling. Read mapping depth and quality were assessed using QualiMap v.2.1.1 (García-Alcalde et al. 2012). GATK v.4.1.1.0 (McKenna et al. 2010) was used to call variants across the samples. *HaplotypeCaller* was implemented to generate intermediate per-sample genotype likelihoods, which were then merged and jointly genotyped. Biallelic SNPs were selected and subsequently hard filtered (QD2 > 2, QUAL > 30, SOR < 3, FS < 60, MQ > 40, MQRankSum > -12.5, ReadPosRankSum > -8.0) using standard hard filtering parameters according to GATK Best Practices recommendations (https://gatk.broadinstitute.org/) (DePristo et al. 2011).

Our primary focus was the native samples from this dataset, which were sequenced at a higher depth than samples from Helge å. This native sample dataset was filtered to exclude sites with mapped read depth either larger than double, or smaller than one-third of the average aggregated read depth (max read depth: 2028 ×; min read depth: 338 ×), which in addition to filtering out regions of spurious mapping also excluded the mitochondrial sequence. To further filter out low complexity or repetitive regions where reads may be falsely mapped, a mappability mask file was constructed using the SNPable Regions pipeline (http://lh3lh3.users.sourceforge.net/snpable.shtml). Briefly, the de novo genome reference fasta sequence was split in overlapping 150-mers and mapped back onto the reference genome. The result is a fasta-like mask file with a quality score from 0 to 3 for each position in the genome, where 0 indicates very poor mapping ability and 3 that the majority of overlapping 150-mers were mapped uniquely without mismatches. The mask file was converted to a bedfile using msmc-tools (Schiffels and Durbin 2014), and all SNPs in repeat regions were removed from the dataset using BCFtools v.1.9 (Li et al. 2009). BCFtools was also used to filter out SNPs with minor allele frequency lower than 0.01, SNPs where less than 90 % of the samples were genotyped, and all SNPs on scaffolds shorter than 10 kilobases (kb). The resulting high-quality SNP dataset, hereafter referred to as the “native SNP-set”, was used in downstream analyses on the native population.

Samples from the reintroduced, admixed Helge å population were sequenced at a lower sequencing depth compared to the native samples. In order to account for this, we subsampled reads from the native samples in order to get similar coverage across all individuals for comparison to Helge å. Seqtk v.1.2 (https://github.com/lh3/seqtk) was implemented to subsample forward and reverse raw fastq reads separately, with number of reads set to 9,400,000 (approximately the number of reads in the sample with lowest read coverage divided by two) and random seed set to the same value (-s100) in both command executions to sample paired reads. The subsampled reads were then processed, mapped, and filtered in the same way as the full dataset, as described above, resulting in a second dataset containing samples from all four populations, hereafter referred to as the “downsampled SNP-set”.

### Mitochondrial haplotype analysis

To assess mitochondrial diversity and the phylogeny of the Swedish wels catfish populations, we assembled the mitochondrial genome of all individuals included in the study against a reference mitochondrial genome. Using a wels catfish mitochondrial sequence from Greece (Vittas et al. 2011; NCBI accession nr: NC_014261.1), similarity search with *blast* v.2.9.0 + (Altschul et al. 1990) identified an 18,402 bp scaffold of our de novo genome containing the mitochondrial genome (the last ~2000 bp of the Greek mitochondrion were contained twice in our scaffold, likely explained by misassembly due to the circular shape of the mtDNA molecule). All reads that mapped to the identified region for each sample were extracted, converted back to fastq format with SAMtools, then mapped to the Greek mitochondrial sequence reference using the same pipeline as described above for the nuclear data, with the exception that insertions and deletions (indels) were kept as variants, and no filters were applied regarding minor allele frequencies or missing genotypes. Consensus sequences in fasta format were then extracted for all samples using BCFtools, aligned using the MAFFT v.7 online alignment tool (Katoh et al. 2019), which was also used to convert to nexus format in order to construct a minimum spanning network in PopART v.1.7 (Leigh and Bryant 2015).

### Genetic diversity estimates

Diversity statistics from the filtered SNP sets were estimated by VCFtools 0.1.16 (Danecek et al. 2011). The proportion of heterozygous sites (*H*) was calculated as the number of heterozygous sites divided by the total number of SNP sites (across all populations) for all individuals. To test whether there were any significant differences in *H* between the populations, we ran a one-way ANOVA followed by a post-hoc Tukey’s test in R v. 3.6.1 (R Core Team 2017). Nucleotide diversity (π) was calculated in windows of 10 kb across the genome for each population and the genome-wide average was reported. The proportion of polymorphic sites (*P*) was calculated for each population. For this purpose, we calculated π for each SNP in each population to get information on whether the site was variable (π > 0 means that more than one allele is present). The number of sites where π was larger than 0 was then divided by the total number of SNPs (across all populations) to get an estimate of the total variance that exists in each population.

To adjust for the differing sample sizes between the native populations (*N* = 20 per population) and Helge å population (*N* = 6) when these calculations were performed on the downsampled SNP-set, we performed 100 random resamplings of six individuals from each native population when calculating *P*, π and *H*, and report the average values (± sd). For the downsampled *H* values, we ran one-way ANOVA tests in R for all the 100 re-samplings and post-hoc Tukey’s tests for all of those where the ANOVA P-value was lower than 0.05.

### Population structure

To assess the genetic structure among and within the three native populations, and estimate the genetic ancestry of the admixed population of Helge å, we performed a principal components analysis (PCA) in PLINK and a genetic ancestry analysis in the software ADMIXTURE (Falush et al.^ 2013^). The PCA was conducted using the default settings, retaining the first 20 PCA components. ADMIXTURE estimates the genetic ancestry of the samples in a dataset, based on a specified number of populations (K). ADMIXTURE was run for values of K from one to six and set to run with cross-validation (CV) outputting a CV error value for each individual K-value. The CV error is an estimate of the predictive accuracy, where the K with the lowest CV error can be interpreted as the most probable number of true genetically distinct populations in the dataset (optimal K-value). To avoid bias due to linkage, both datasets were pruned for strong linkage prior to using PLINK, removing SNPs with pairwise r^2^ values higher than 0.5 in sliding windows of 50 SNPs moving stepwise with five SNPs at a time across the genome. Genetic differentiation between population pairs was estimated in VCFtools using Weir and Cockerham’s *F*_ST_ estimate in non-overlapping windows of 10 kb. The genome-wide average was then calculated for the weighted pairwise *F*_ST_ values. To investigate within-population substructure within Möckeln and Emån, each population was randomly subsampled into two populations of equal size and pairwise *F*_ST_ estimated by VCFtools for 100 iterations, and compared to the pairwise *F*_ST_ estimated from the sample divisions underlying the substructure identified by ADMIXTURE.

### Runs of homozygosity

The presence of long autozygous stretches in the genome, termed runs of homozygosity (ROH) (Gibson et al. 2006), serve as an estimate of recent and ancient inbreeding. PLINK was used to estimate ROHs in both datasets, such that the length of a homozygous stretch needed to exceed 100 kb to be called a ROH, at least 50 homozygous SNPs per ROH (minimum density of one SNP per 50 kb), one heterozygous SNP and five missing genotypes were allowed per scanning window, and consecutive SNPs more than 1000 kb apart could not be in the same ROH. Using the output from this analysis, we calculated the fraction of each individual genome that fell inside ROH segments of > 100 kb and > 1000 kb, separately (*F*_ROH>100_ and *F*_ROH>1000_, respectively). In order to do this, we first calculated the ROH analysis coverage across the genome for both these ROH lengths i.e. the length of the genome with sufficient SNP density for ROH detection, by constructing an artificial sample with homozygous genotypes across all SNPs in the dataset, following Meyermans et al. (2020). The aggregated length of ROH segments in this simulated homozygous sample then served as the denominator when calculating the *F*_ROH_ values. To test for significant population effects on the prevalence of ROHs, we ran a one-way ANOVA in R on the *F*_ROH_ values, followed by a post-hoc Tukey’s test.

## Results

### De novo wels catfish genome assembly

Supernova produced a reference de novo genome assembly with a total length of 901 Mb, corresponding to 89 % of the total genome length of 1.01 Gb as estimated by the software. Scaffold N50 and L50 values were 3.8 Mb and 65, respectively, and the longest scaffold measured 22.2 Mb. After removal of scaffolds shorter than 10 kb, the length of the genome used in downstream analyses was 753.7 Mb.

### Genomic diversity

From all 66 samples, a total of 6,524,000,000 raw sequenced reads were obtained, averaging 106,710,000 per native sample and 20,230,000 per Helge å sample. After mapping and filtration, mean read coverage on the reference genome was 16.9 x in the native dataset. A total of 1,891,836 biallelic SNPs were called, of which 546,771 high quality SNPs remained after full filtration. The average mapped read coverage in the down-sampled SNP set was 2.99x. For this dataset, 1,226,788 SNPs were initially called and 236,435 SNPs remained after quality filtration.

The highest genetic diversity of the three native populations was found in Emån, according to all three estimates (Table 1). The population-level estimates π and *P* were 2.49 × 10^−4^ and 0.73, respectively, in Emån, while Båven and Möckeln showed similar slightly lower values of π = 2.13 × 10^−4^ and *P* = 0.61, and π = 2.16 × 10^−4^ and *P* = 0.61, respectively. The average *H*, which indicates diversity on an individual level, was also highest in Emån at 0.24 (±0.03), higher than both Båven at 0.20 (±0.01) and Möckeln at 0.20 (±0.02). A one-way ANOVA showed that there were statistically significant differences in *H* between the populations (F_(2,57)_ = 19.36, *P* < 0.001), and the post-hoc Tukey’s test revealed significant differences between Emån and the two other native populations (*P* < 0.001 in both comparisons) while Båven and Möckeln did not differ significantly (*P* = 0.93).

**Table 1:** Estimates of genetic diversity calculated from the native and down-sampled SNP-set, including the average nucleotide diversity (π); number of heterozygous sites (H); and proportion of polymorphic sites (P).

Population	π	*H*	*P*
Native dataset			
Båven	2.13 × 10^−4^	0.202 (±0.011)	0.615
Emån	2.49 × 10^−4^	0.236 (±0.026)	0.726
Möckeln	2.16 × 10^−4^	0.204 (±0.017)	0.610
Down-sampled dataset			
Båven	1.10 (±0.009) × 10^−4^	0.091 (±0.003)	0.346 (±0.003)
Emån	1.04 (±0.011) × 10^−^^4^	0.096 (±0.009)	0.393 (±0.010)
Möckeln	1.07 (±0.027) × 10^−4^	0.094 (±0.013)	0.369 (±0.016)
Helge å	0.87 × 10^−4^	0.085 (±0.011)	0.348

Standard deviation (sd) is shown in parentheses, for the down-sampled SNP-set, the sd of the 100 resamplings of the native populations are presented (except for the *H* in Helge å, where between-sample sd is presented).

In the downsampled SNP-set, the diversity estimates of the three native populations were more similar to each other, while the reintroduced population of Helge å showed lower values in all three analyses (Table 1). Also, in this dataset, Emån had the highest *P* (0.39) and *H* (0.096 ± 0.003), while π was highest in Båven (11.1 × 10^−5^ ± 0.1 × 10^−5^). In the majority (84) of the 100 random resamplings of six individuals per native population; however, population did not have a statistically significant effect on *H*.

### Population structure

**Fig. 2:**
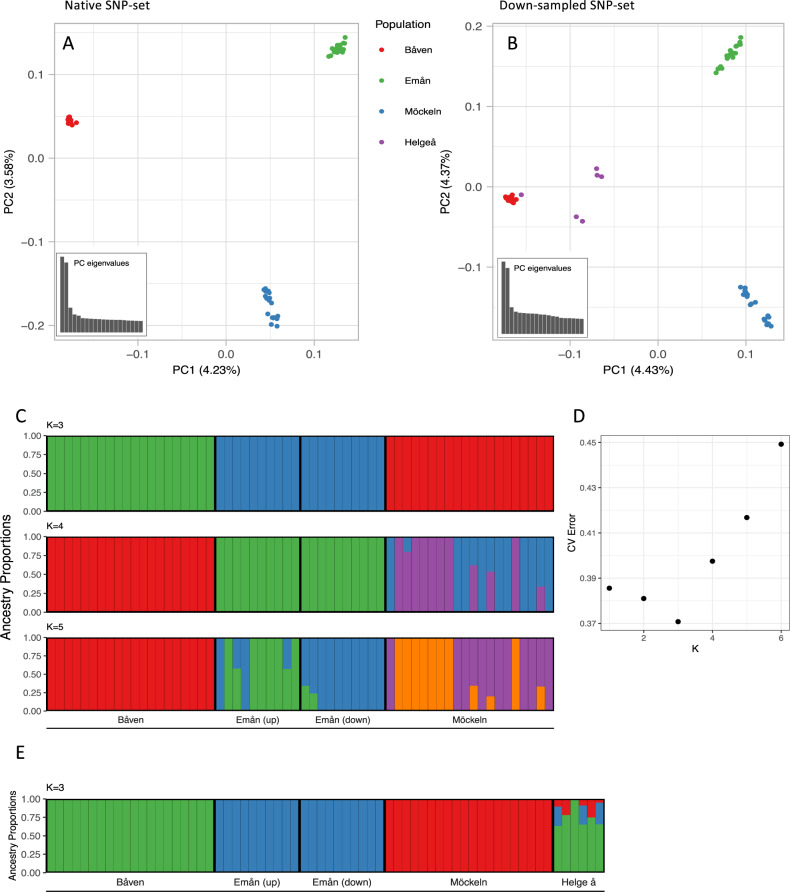
Population structure and admixture among Swedish populations of wels catfish, *Silurus glanis*, inferred from the native SNP-set (data from the native populations of Båven, Emån, and Möckeln) and the downsampled SNP-set (including also the reintroduced population of Helge å). **A** The PCA-analysis of the native individuals shows distinct, non-overlapping, groups based on population origin. **B** The same analysis from the down-sampled SNP-set resulted in similar groups of native individuals, while the Helge å samples sit more central in the plot, but closest to Båven. **C** ADMIXTURE plot showing that the populations are distinct without admixture at *K* = 3, while *K* values of 4 and 5 show some substructure within Möckeln and Båven, respectively. **D** The CV-error values from the ADMIXTURE analysis shows that the lowest CV-error value was found at *K* = 3 (optimal K). **E** The ADMIXTURE analysis on the down-sampled SNP-set shows that the individuals from Helge å are of admixed origin, with the highest proportion of genetic ancestry deriving from Båven.

The PCA revealed clear genetic structure. The native populations formed distinct, non-overlapping clusters in the PCA (Fig. 2A). The first axis, explaining 4.23 % of the variance clearly separated Båven from Emån and Möckeln, while the second axis explained 3.58 % of the variance and clearly separated Möckeln from Emån and Båven. An optimal K of three was found in the ADMIXTURE analyses (Fig. 2D), which assigned samples to genetic clusters corresponding perfectly with the three native populations (Fig. 2C). As K was increased, some substructure became apparent within Möckeln (at *K* = 4) and Emån (at *K* = 5). The structure appearing within Emån at *K* = 5 roughly corresponded to upstream and downstream sampling locations. Genetic differentiation between the native populations was similar across the three pairwise *F*_ST_ estimates. The weighted *F*_ST_ estimate between Båven and Emån was the highest at 0.196, followed by Båven and Möckeln at 0.195 and Emån and Möckeln at 0.170. The pairwise *F*_ST_ estimates across the genome displayed high variance between the populations without any obvious outliers displaying elevated levels of differentiation (Fig. S2). Differentiation within Emån and Möckeln was also investigated using the sample divides identified by ADMIXTURE. Within Emån, mean weighted *F*_ST_ was 0.038, while within Möckeln it was 0.022 (Fig. S3). These *F*_ST_ estimates were higher than those values derived by estimating differentiation from random subsampling of the populations (Fig. S4).

The individuals sampled in Helge å had the highest proportion of genetic ancestry from Båven, as visualized in the PCA of the downsampled SNP-set (Fig. 2B). Samples from Helge å generally grouped towards the center of the PCA plot, but in closer proximity to the Båven samples. One sample grouped together with samples from Båven. ADMIXTURE assigned the highest proportion of the Helge å samples to Båven (Fig. 2E). Across all samples, 74.6% of the genetic origin was assigned to Båven, 13.5% to Emån, and 11.9% to Möckeln.

### Runs of homozygosity

**Fig. 3:**
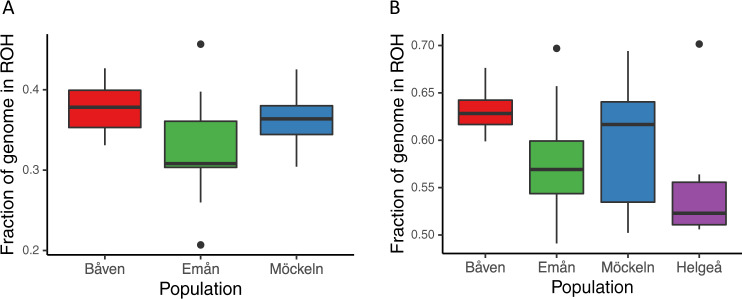
Runs of homozygosity in catfish populations. Proportion of the genome contained in ROH segments longer than 100 kb (*F*_ROH>100kb_) inferred from the native (A) and down-sampled (B) SNP-set.

The ROH analysis coverage from the native SNP-set was 647 Mb and 605 Mb for ROH lengths of > 100 Kb and > 1000 Kb, respectively, such that 98.7 % of the genome on scaffolds > 100 kb and 99.5% on scaffolds over 1000 kb were sufficiently covered by SNPs for ROHs to be called (i.e. lack of ROHs are not caused by insufficient SNP density). A large genomic proportion of native individuals consisted of ROH segments (Fig. S5), although almost exclusively shorter than 1000 Kb. *F*_ROH>100kb_ spanned from 0.21 to 0.46, with the population averages of 0.33 ± 0.05 in Emån, 0.36 ± 0.03 in Möckeln, and 0.38 ± 0.03 in Båven (Fig. 3A). In line with average *H* values, Emån had significantly lower *F*_ROH>100Kb_ values than both Båven (*P* < 0.001) and Möckeln (*P* = 0.008) while the difference between Båven and Möckeln was non-significant (*P* = 0.63), as found through a post-hoc Tukey’s test following a one-way ANOVA showing significant population differences (F(2,57) = 8.99, *P* < 0.001). Only four ROH segments over 1000 kb were found distributed across three individuals, all of which were just over 1000 kb in length. Thus, we did not specifically calculate *F*_ROH>1000kb_ as it was zero or close to zero across all individuals.

We also ran a ROH scan on the down-sampled SNP-set, to infer the relative abundance of ROHs in Helge å compared to the native populations. ROH analysis coverage changed only marginally compared to the native SNP-set, resulting in analyzable lengths of 641 Mb and 601 Mb for ROHs > 100 Kb and ROHs > 1000 kb, respectively. As a consequence of the down-sampling, the heterozygosity was reduced in the native populations, leading to a larger proportion of the genome detected as ROH segments (Fig. 3B). The one-way ANOVA test showed that a significant population effect on ROHs remained after downsampling (*F*_(3,62)_ = 5.70, *P* = 0.002), and the post-hoc Tukey’s test revealed that the samples from Emån still had significantly lower *F*_ROH>100KB_ values than Båven (*P* = 0.006) while the other comparisons among the native populations were non-significant (*P* > 0.17). The samples from Helge å had on average lower *F*_ROH>100KB_ than the native populations and were significantly differentiated from Båven (*P* = 0.008) but not from Emån and Möckeln (*P* > 0.23).

### Mitochondrial diversity

**Fig. 4:**
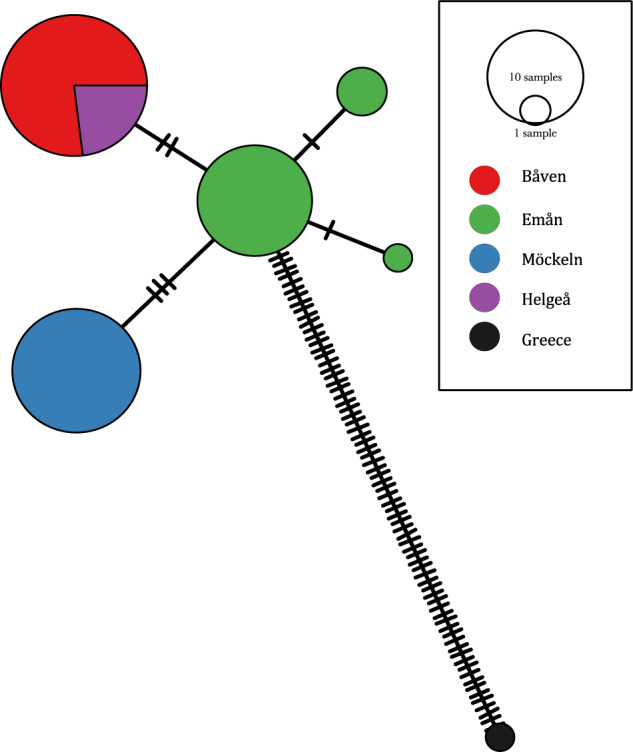
Mitochondrial haplotype network showing the five segregating haplotypes in the Swedish populations and their distance to the Greek reference. The ticks in the lines between the haplotypes represent a one-nucleotide difference.

All samples produced a mitochondrial haplotype with length of 16,524 bp. Overall, the mitochondrial diversity was low, with only five haplotypes found across all samples (Fig. 4). Only seven SNPs were found (no indels) between these haplotypes, and no haplotypes were shared across the native populations. In line with the observations from nuclear genomic diversity estimates, we found the highest mitochondrial diversity in Emån with three haplotypes present. The most common mitochondrial haplotype was carried by 16 individuals, with the next two most common being present in just three and one individuals, respectively. The latter two were each separated by a single nucleotide substitution from the most common one. Individuals from Möckeln and Båven all possessed a single population-specific haplotype, differing by three and two nucleotide substitutions from Emån, respectively (Fig. 4). All samples from Helge å possessed the Båven mitochondrial haplotype.

## Discussion

Recent applications of population genomics have begun to address conservation and management issues (Hohenlohe et al. 2020; Hohenlohe and Rajora 2020 and references therein). One such application is to assess and quantify genetic diversity and levels of inbreeding in species at a high risk of extinction. A recent study on mammals showed that such species displayed lower levels of genetic diversity (Zoonomia Consortium 2020). Since high throughput sequencing techniques and bioinformatic analyses have become more widely applied, more affordable, and more adaptable, there has been an ongoing debate as to how whole genome sequencing will aid in conservation planning and whether such knowledge will be useful for practical conservation action (Allendorf et al. 2010; Garner et al. 2016; McMahon et al. 2014; Shafer et al. 2015; Shafer et al. 2016). Bridging the gap between geneticists and conservation practitioners has therefore become a main focus (Taylor et al. 2017). A pertinent question in this endeavor is whether inferences made from whole-genome sequencing approaches are worth the increased costs and expertize needed when compared to more conventional genetic analyses such as targeted sequencing and marker-based methods like microsatellite analyses (e.g., Brandies et al. 2019).

Here, we show that many of the inferences made in a previous microsatellite-based analysis (Palm et al. 2019) remain robust and are hence corroborated by our whole-genome re-sequencing approach. This is to our knowledge one of the first intraspecific comparisons of the same study populations in which traditional marker-based genetic study can be directly compared with genomic study. We also show that some of the findings with important conservation consequences may be altered and that the whole genome approach allows deeper understanding of some key parameters of relevance for conservation. This is especially true for inferences about possible past and present inbreeding, which can be made from resequencing data but are more obscure in a microsatellite study. Below we discuss our findings and discuss their resemblance and differences to the previously published microsatellite analyses (Palm et al. 2019). This is no way in disregard of the quality of previous work but as an example how different methods and analytical tools may or may not arrive at different conclusions.

### Genomic diversity

We found that the population of catfish from Emån displayed slightly higher estimates of genetic variation than the two other extant native Swedish populations of Båven and Möckeln, regardless of whether diversity was estimated as nucleotide diversity (π), heterozygosity (H) or proportion of polymorphic loci (P). This is in contrast to Palm et al. (2019) who reported the highest diversity in Båven followed by Emån and then Möckeln. This difference may be indicative of the uncertainty of basing genetic diversity estimates on a few highly polymorphic loci when comparing populations. However, both resequencing and microsatellite data indicate similar levels of genetic variation among native Swedish catfish populations and we suggest any fine-scale differences among populations should be regarded with caution.

It is clear from our data and the previous microsatellite study (Palm et al. 2019) that Swedish catfish populations contain much lower levels of genetic variability than populations from the main distribution in central and eastern Europe (Krieg et al. 2000; Triantafyllidis et al. 2002). In particular, mitochondrial haplotype diversity found in this study is very low, with only three haplotypes in Emån and unique single haplotypes in the other two populations. Furthermore, individuals from the reintroduced population in the lower part of Helge å all had the Båven mitochondrial haplotype. The low mitochondrial diversity could indicate a severe founder event during colonization of Sweden. Dispersal from mainland Europe to Scandinavia would have been possible when the Baltic Sea was as a freshwater lake, sealed off from the marine oceans (approx. 13,000–9500 B.C. and 8800–7800 B.C.). The severity of this founder event may also explain the general low levels of genetic diversity in native Swedish catfish.

### Population structure and genetic differentiation

The microsatellite study and our resequencing approach arrived at similar conclusions regarding population structure. Admixture analyses showed clear population differentiation among the three extant native catfish populations in Sweden irrespective of data type, whether whole genome-wide SNPs, or allelic variation at 10 microsatellite loci was used (our Fig. 1, Fig. 3 in Palm et al. 2019). *F*_*ST*_ scans showed regions of extremely high differentiation and high variability with no obvious peaks of elevated differentiation across the genome between the native populations (Fig. S2). Such signatures likely reflect a history of population isolation and genetic drift. This is compatible with a scenario where Sweden was colonized by a limited number of founders after the last glaciation, a founder effect that would have led to lost genetic variation. Genetic drift has since been further exacerbated by fragmentation and isolation in the remaining native populations.

Palm and coworkers (2019) report population structure between the upper and lower parts of river Emån. This result could only be replicated in Admixture with our genome-wide dataset by allowing for more than the optimal *K* = 3 clusters. In fact, we detected substructure within Lake Möckeln (*K* = 4) prior to Emån (*K* = 5) (Fig. 2C). Using *F*_*ST*_ scans, we were able to observe higher than expected differentiation within Emån, implying that some population differentiation may be developing between the upper and lower parts of this river. The genetic clusters that are detected in Emån at *K* = 5 are separated by two hydroelectrical power plants that were built in 1904, where fish-ways were established only in the early 2000s (Calles and Greenberg 2005). This part of the river is the steepest, with a total drop of 14 m (upper Finsjö 5.5 m and lower Finsjö 8.5 m) (Palm et al. 2008). It cannot be ruled out that prior to the dam being constructed, this site in the river was also a natural barrier for the migration of catfish. In Möckeln, there are no physical barriers separating the samples that could explain the observed structure at *K* = 4. It is possible that this weak differentiation is instead explained by for example behavioral mechanisms and small population size, as catfish are known to show strong site fidelity (Carol et al. 2007), but further studies are needed to resolve this as we could only speculate at this stage. Whether the differentiation patterns we observe reflects neutral processes (isolation by distance) or local adaptation is unclear. But considering the suspected small size of catfish populations, it is likely that signals of local adaptation will be highly confounded by genetic drift. Genome-wide association studies could be useful here to investigate potential signals of local adaptation. Thus, while we do not rule out the presence of preliminary substructure in Emån and potentially also in Lake Möckeln, we caution against conserving different clusters within the same water basin. Such a scenario should only be considered if strong additional evidence of local adaptation and natural barriers to gene flow becomes available (Fraser and Bernatchez 2001).

### Runs of homozygosity and Inbreeding

With our resequencing approach, we could detect short ROHs in the native populations, indicative of a common past bottleneck (Ceballos et al. 2018). In addition to the observed low genetic diversity, this result is consistent with a founder effect coinciding with the colonization of Swedish waterways after the last glaciation. We failed, however, to find long ROHs indicative of recent inbreeding. Although we interpret this result with caution as our reference assembly and resequencing approach may have hampered our possibilities to find long ROHs, we also note that signs of recent inbreeding (positive F_IS_ and extensive linkage disequilibrium) were only detectable in one of the populations (Emån) using microsatellites (Palm et al. 2019). This strongly suggests that recent inbreeding is low or absent in the three native populations of Swedish catfish. Also of note is that the absolute levels of inbreeding (F_ROH_) found in our study are likely inflated in the comparisons involving the reintroduced Helge å population due to the lower quality of data from this population, which required down-sampling the native populations to appropriately allow comparisons. Given the shallow mapped read depth in this dataset, homozygosity is likely overestimated and should not be interpreted as absolute values. Instead, the relative levels of inbreeding across the populations should be considered, and as expected, the recently established reintroduced population (with founders from at least two native populations, see below) exhibited less inbreeding.

### Reintroduced population of Helge å

Lake Möckeln is a part of the Helge å water system and the river drains to the Baltic Sea. The lower part of this river is completely sealed off by a hydroelectric power plant. Catfish were absent from this part of the river, until approximately 20 years ago when restocking was initiated using fish from all three native populations (Svensson et al. 2013). As expected, and in line with the microsatellite study (Palm et al. 2019), our results revealed an that this artificially reintroduced population was of admixed ancestry, with the largest genetic material being of Båven origin. Furthermore, our mitochondrial data suggests a predominant contribution of Båven matrilines with all mt-DNA profiles identical to the one haplotype present in Båven. Thus, despite efforts to admix the reintroduced population, the current realized admixture was lower than expected. In the early 2010s, additional stocking was performed with approximately 90 ex-situ bred juveniles (full-siblings), genetically originating from Möckeln (Svensson et al. 2013). Sexual maturity in the Swedish populations occurs at around 8–12 years (Nathanson 1987), so it is unlikely that these individuals have yet contributed to the gene pool. We highly recommend surveying the genetic composition of this population over the next decade, in order to monitor contributions from Möckeln and plan conservation actions for increased genetic diversity.

Our estimates of genetic diversity suggest the reintroduction has further reduced already low levels of genetic variation. This is a result contradicted by the microsatellite study, which suggested higher levels of genetic variation in Helge å when compared to the native populations. We cannot completely exclude the possibility that the lower sequencing depth in the Helge å samples may be the reason for the lower observed diversity. We attempted to rectify this by down-sampling our native data set to the same sequencing depth and number of individuals when making comparisons to the native populations. When subsampling for the same sample size we could not detect any statistical differences among Helge å and the native populations. This lack of significance is likely explained by a lower power of the statistical test. Regardless, we failed to find evidence of increased genetic variation in the reintroduced population, which is in line with our admixture analyses that indicated a large fraction of the genomes of Helge å individuals are of Båven origin.

The reduced fraction of the genomes in ROHs observed in Helge å does suggest that, as expected, the level of admixture obtained has been sufficient to reduce tracts of homozygosity in the Helge å fish. This result is somewhat at odds with the failure to detect higher genetic diversity in the Helge å fish. Further resequencing of more fish at greater depth will be needed to resolve whether the reintroduction will lead to increased levels of genetic diversity over time.

### Overall comparison of microsatellites and resequencing

Overall, the two approaches (10 microsatellites typed in >1000 fish vs. whole-genome resequencing of slightly more than 60 fish) yielded remarkably similar results and conservation implications. Both approaches suggest marked genetic differentiation among the native populations, reduced genetic variation in isolated Swedish wels catfish populations as expected from a postglacial founder event, low levels of recent inbreeding in the native populations, and a major contribution of one of the founder populations in the restocked population of Helge å. Thus, it may be asked: what motivates a whole-genome resequencing project when the established microsatellite approach yielded similar results? First of all, we argue it is reassuring that different methods arrive at similar results and conservation recommendations. Different approaches have their different costs and benefits. A marker-based study, such as one based on microsatellites, yields data on a few loci from many fish while a resequencing approach yields many loci from fewer fish. Which is more reliable will depend on the specific research question: in this particular case, questions, results, and interpretation mostly converge. There may be circumstances, however, when one approach is more suitable than the other (Brandies et al. 2019). One such aspect relates to estimates of inbreeding in which a resequencing approach yields better genomic coverage and allows for more in-depth studies of past and present inbreeding events (Ceballos et al. 2018). Another aspect relates to attempts to identify regions and loci under putative selection to find evidence of local adaptation (Savolainen et al. 2013). It is our conviction that a resequencing approach is a cost effective and adequate way forward in conservation genetics and significantly contributes to the global field of biodiversity genomics. The cost of the present study was in the order of 10,000 USD all included. While benefitting from access to high-performance computer resources (Uppsala Multidisciplinary Center for Advanced Computational Science) and sequencing facilities at a major research institution (SNP&SEQ Technology Platform in Uppsala), we would not have been able to conduct a microsatellite study at a competitive cost.

### Conservation implications

In line with Palm et al. (2019), our results show low genetic diversity within, and high genetic differentiation among the Swedish native wels catfish populations. Low genetic diversity increases the vulnerability of populations to sustained environmental changes, heightening extinction risks (Frankham et al. 2010). Increasing and maintaining genetic variation in these populations are important aims for catfish conservation in Sweden. The percentage of variable loci (*P*) indicates how much of the total variation is present in each of the subpopulations. The values of *P* indicate that the native populations each hold 62–73% (and thus lack 27–38%) of the total standing variation, such that genetic diversity could be substantially increased by translocations of individuals between populations. Caveats of translocations are risks of introducing alien diseases or parasites. One such example is a parasitic worm that has been reported on catfishes in Möckeln but not in Emån and Båven (Svensson et al. 2013). If populations are highly diverged, there is also a risk of outbreeding depression (Frankham et al. 2011; Tallmon et al. 2004). Considering the seemingly shallow absolute divergence between the Swedish populations indicated by the mitochondrial haplotype network, the risks of outbreeding depression are likely low, but local adaptation cannot be ruled out. Importantly, the reintroduced population in Helge å provides a significant opportunity to investigate the effects of mixing the native populations. Following this population over a longer time period and performing deeper sequencing to study genetic diversity more reliably would be extremely valuable to predict the effects of translocations of individuals among the native populations. While establishing permanent connections between the native populations seems unrealistic today due to the large geographical distances that separate them, the apparent incipient population structure in Emån, and possibly also in Möckeln, highlights the need for connectivity within the populations. Until the effects of translocations can be further evaluated, stimulating connectivity and population growth within the native populations are important tasks to ensure long-term viability of the wels catfish in Sweden.

## Supplementary information

Supplementery Figures

Supplementery Table

## Data Availability

Raw reads from the Chromium 10x linked-read sequencing (one individual), as well as the Illumina short-read whole-genome sequencing (66 individuals) are available via the Sequence Read Archive (SRA, https://www.ncbi.nlm.nih.gov/sra) under the accessions SRX8712101-SRX8712120 (BioProject accession PRJNA645600).
