# Supplementary material for: Whole genome sequencing reveals high differentiation, low levels of genetic diversity and short runs of homozygosity among Swedish wels catfish
Source: Heredity (Edinb). 2021 May 7;127(1):79–91. doi: 10.1038/s41437-021-00438-5 (PMC8249479; doi:10.1038/s41437-021-00438-5)
Supplement: Supplementary file 1 — Supplementery Figures [file 41437_2021_438_MOESM1_ESM.pdf]

Whole genome sequencing reveals high differentiation, low levels of genetic diversity and short runs of homozygosity among Swedish wels catfish

Axel Jensen[1], Mette Lillie[1,2*], Kristofer Bergström[3], Per Larsson[3], Jacob Höglund[1]

[1] Department of Ecology and Genetics, Animal Ecology, Uppsala University, Uppsala, Sweden

[2] Department of Biological and Environmental Sciences, University of Gothenburg, Gothenburg, Sweden

[3] Department of Biology and Environmental Science, Faculty of Health and Life Sciences, Linnaeus University, Kalmar, Sweden

[*] Corresponding author, mette.lillie@imbim.uu.se

Supplementary Figures

Contents:

Figure S1. Approximate distribution for the Swedish populations of wels catfish sampled in this study, highlighting water drainages	Page 2
Figure S2. Weighted mean F_{ST} in 10kb windows between Swedish native population pairs	Page 3
Figure S3. Weighted mean F_{ST} in 10kb windows across the longer scaffolds of the catfish assembly within Emån and Möckeln	Page 4
Figure S4. Distribution of mean weighted F_{ST} values across the catfish assembly	Page 5
Figure S5. The distribution of runs of homozygosity (ROHs) on scaffold 19	Page 6

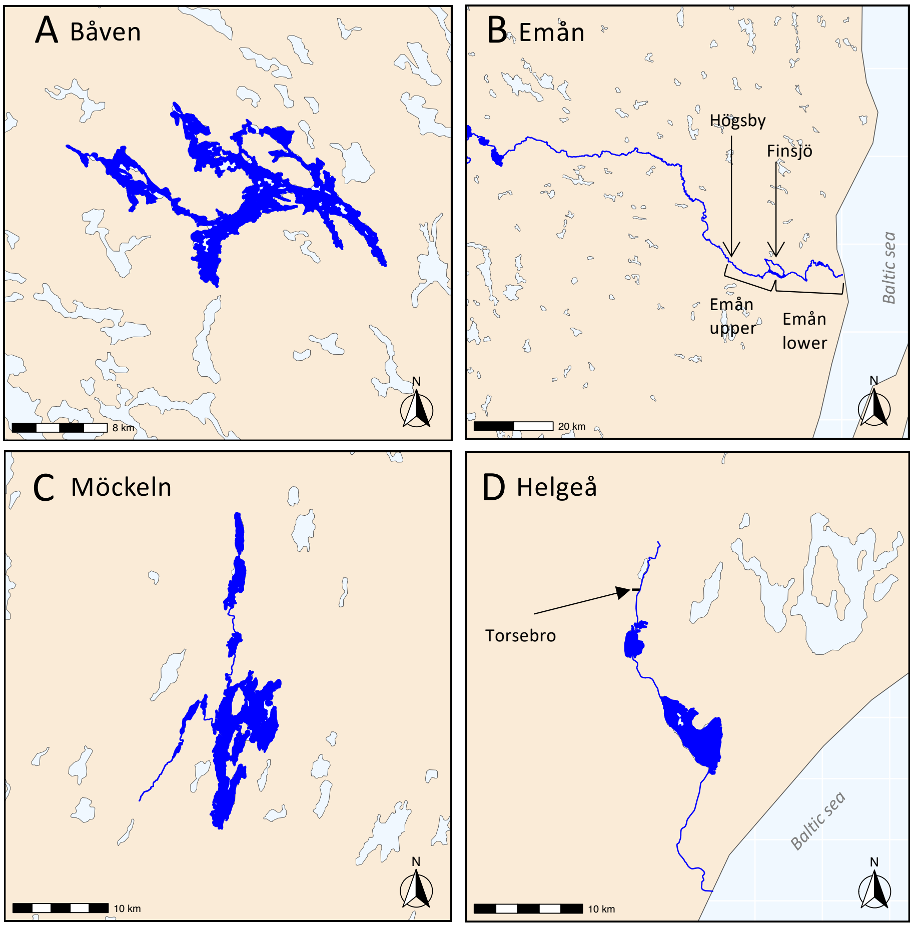

Figure S1. Approximate distribution for the Swedish populations of wels catfish sampled in this study, highlighting water drainages, including the extant populations of Lake Båven (A), River Emån (B), Lake Möckeln (C), and the reintroduced population in River Helgeå (D). The catfish in Emån (B) has previously been suggested to represent two distinct populations (Emån upper and Emån lower), separated by two hydropower plants and possibly the steep nature of the river around Finsjö. Catfish are known to occur in Emån between the river mouth and Högsby. Upstream from Högsby the distribution is not well known. In Helgeå (D), reintroductions have been performed downstream Torsebro, where a hydropower plant is present making upstream migration largely impossible.

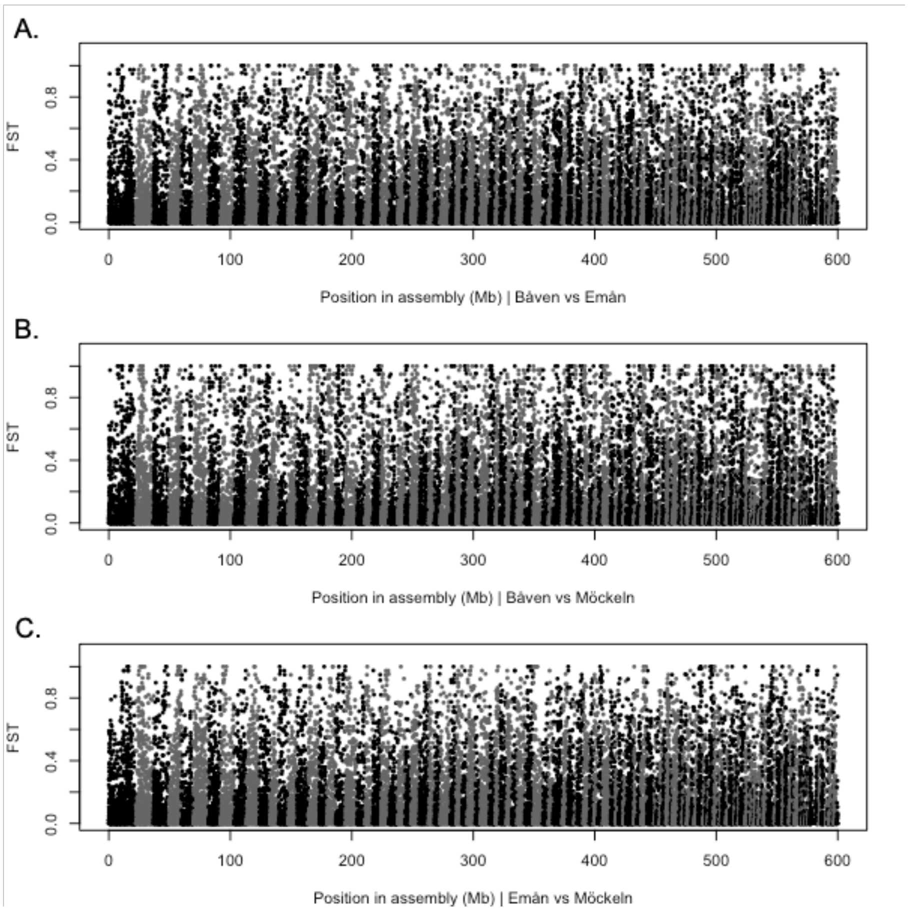

Figure S2. Weighted mean F_{ST} in 10kb windows between Swedish native population pairs, including (A) Båven versus Emån, (B) Båven versus Möckeln and (C) Emån versus Möckeln, across the longer scaffolds of the catfish assembly.

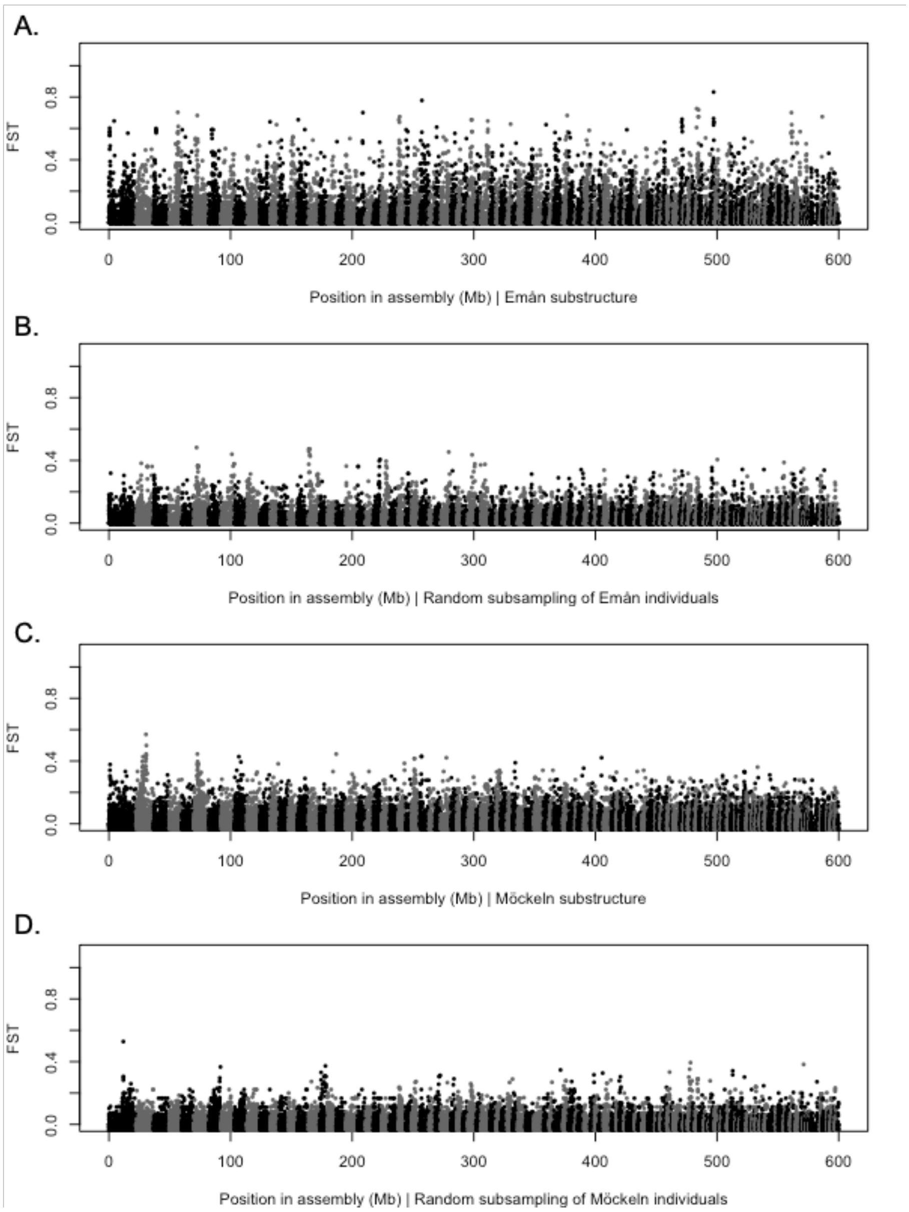

Figure S3. Weighted mean F_{ST} in 10kb windows across the longer scaffolds of the catfish assembly within Emån and Möckeln to investigate developing substructure. (A) F_{ST} scan between upstream and downstream Emån samples; (B) F_{ST} scan of Emån samples randomly assigned to population; A) F_{ST} scan between Möckeln samples with population assignment derived from Admixture; (B) F_{ST} scan of Möckeln samples randomly assigned to population.

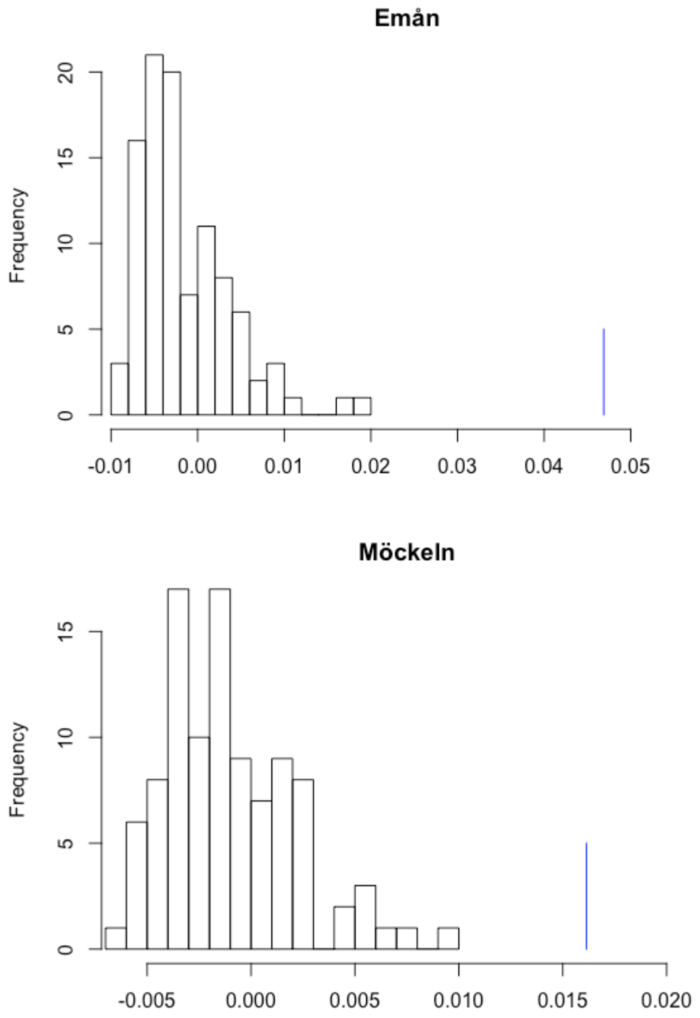

Figure S4. Distribution of mean weighted F_{ST} values across the catfish assembly when randomly sampled in populations of Emån (upper) and Möckeln (lower). Blue line indicates mean weighted F_{ST} values of the inferred population substructure.

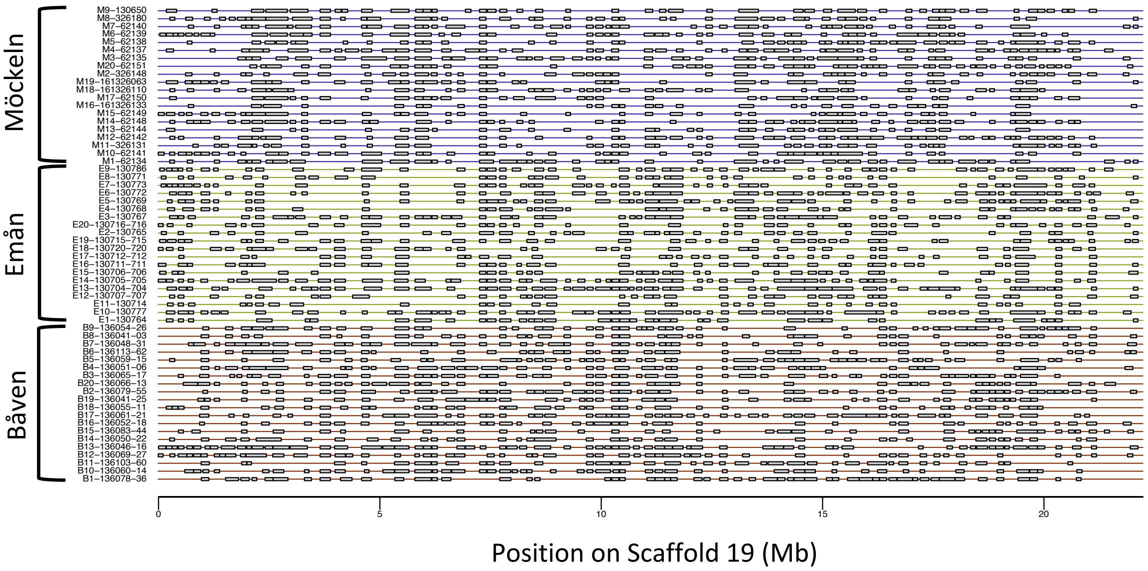

Figure S5. The distribution of runs of homozygosity (ROHs) on scaffold 19 (longest scaffold in the wels catfish assembly). Each horizontal line represents one individual's genomic region, colored by population (red = Båven, green = Emån, blue = Möckeln). The grey rectangles represent ROHs.
